# Prevalence of rifampicin resistant tuberculosis among pulmonary tuberculosis patients In Enugu, Nigeria

**DOI:** 10.4314/ahs.v22i2.18

**Published:** 2022-06

**Authors:** Amara Ulasi, Ndubuisi Nwachukwu, Reginald Onyeagba, Solomon Umeham, Anuli Amadi

**Affiliations:** 1 Abia State University Faculty of Biological and Physical Sciences, Animal and Environmental Biology; 2 Abia State University, Microbiology; 3 South East Zonal TB Reference Laboratory, Amachara, Umuahia, Nigeria, Microbiology

**Keywords:** Multidrug Resistant Tuberculosis, Rifampicin resistance, Gene Xpert, Drug resistance

## Abstract

**Objective:**

We determined the prevalence of rifampicin resistance in pulmonary tuberculosis patients in Enugu Nigeria.

**Methods:**

A prospective hospital-based study involving 1300 presumptive multidrug-resistant tuberculosis patients was conducted in Enugu between April 2017 and 31st March, 2019.

Participants age ranged from 15 years and older and each submitted one sputum specimens Sputum specimens were analyzed using the Gene Xpert MTB/RIF assay to detect resistance to rifampicin according to manufacturer's protocol.

**Results:**

The prevalence of rifampicin resistant tuberculosis was 6.8% (95% CI: 5.5- 8.3). Rifampicin resistance was significantly higher in males (9.0%) than females (4.2%) (P = 0.036< 0.05). Most of the cases were seen in the age group 35–44 years (28.4%). Prevalence of rifampicin resistant tuberculosis was 2.7% in treatment naive (new) patients and 4.1% in patients on anti-tuberculosis therapy (previously treated).

**Conclusion:**

The prevalence of rifampicin resistant tuberculosis in Enugu was high. Rifampicin resistance in treatment naive (new) patients was also high. This study therefore highlights that active transmission of Multidrug-resistant tuberculosis among young males could be on-going.

## Introduction

Drug resistant tuberculosis (DR-TB) is a major public health problem that threatens progress made in tuberculosis control worldwide.^1^

Furthermore, the rapid spread of Rifampicin - or Multidrug-Resistant Tuberculosis (MDR/RR-TB) especially in new tuberculosis patients is challenging the effectiveness of tuberculosis control in many low-income countries.^2^ Nigeria is one of the countries included among the 30 high burden countries for TB, TB/HIV and DR-TB.^3^ The World Health Organization estimates that the proportion of patients with MDR/RR-TB is 4.3% among new cases and 25% among previously-treated cases in Nigeria.^3^

Drug resistance results from genetic mutation in specific genes, inadequate or poorly administered treatment regimen and weak services programmes that lead to delayed detection.^4^,^5^ Rifampicin is one of the most important anti-tuberculosis antibiotics; it exerts its bactericidal activity by inhibiting the early steps of gene transcription by binding to the β-subunit of RNA polymerase (rpo) encoded by the rpo gene.^6^ Its inclusion in the anti-TB regimen has shortened the duration of tuberculosis treatment.^7^ Rifampicin resistance is a precursor to the development of multidrug-resistant tuberculosis (MDR-TB) and a reliable predictor of multidrug-resistance in settings where the prevalence of rifampicin resistant Mycobacterium tuberculosis is high^8^,^9^. Its early detection is essential because of high risk of transmission from person to person and emergence of MDR-TB and extensively drug-resistant tuberculosis (XDR-TB).^3^,^10^

Data from the Federal Ministry of Health (FMOH) shows that only 2, 286 MDR-TB cases out of the estimated 43,829 cases were notified in 2017.^11^ Some previous studies have reported the occurrence of tuberculosis drug resistance in various parts of Nigeria using Mycobacterial culture and drug susceptibility test.^12^–^15^ However, culture and drug susceptibility test method have long turn - around time and patients experience mortality and morbidity and community transmission may increase.

To step up the search for the missing tuberculosis drug resistant cases in Enugu, Nigeria, we used Xpert MTB/RIF technology (Cepheid, Sunnyvale, CA, USA) to determine the prevalence of rifampicin resistant tuberculosis among pulmonary tuberculosis patients as it is a significant factor that contributes to MDR-TB outbreaks.

## Methods

### Study area/Design/Period

A prospective hospital-based study was conducted at 3 hospitals in Enugu, Enugu state, Nigeria between April 2017 and March 2019. All the three hospitals: University of Nigeria Teaching Hospital, Parklane hospital and Annunciation hospital are integrated into the National Tuberculosis and Leprosy Control Programme (NTBLCP). These hospitals are referral centers for other peripheral hospitals in Enugu State. Tuberculosis medicines and services are offered for free at these facilities.

### Study participants

The study comprises of 1300 Presumptive MDR-TB patients who attended these TB facilities for care. They were invited to participate and enrolled at the time of presentation to the clinics. Age of participants ranged from 15 years and older.

Operational definition of presumptive MDR.TB cases^3^ Presumptive MDR.TB cases referred to:

1. Treatment naïve subjects who presented with pulmonary tuberculosis symptoms and were close contacts of DR-TB cases.
2. New smear positive pulmonary tuberculosis patients found positive on 2/3 month follow up examination.
3. Previously treated pulmonary tuberculosis cases

### Method

Socio-demographic and clinical information of the patients were obtained using a structured questionnaire and their hospital records. Each patient submitted about 4ml of sputum on the spot. The sputum specimens obtained was analyzed using the Gene Xpert MTB/RIF to detect -Mycobacterium tuberculosis (MTB) and its susceptibility pattern to rifampicin (RIF). The Gene Xpert MTB/RIF is a fully automated diagnostic molecular test using real-time polymerase chain reaction technology to simultaneously detect MTB and RIF resistance mutation in the rpoβ gene.^16^ A sample reagent was added in 2:1 ratio to unprocessed sputum in 15 ml-falcon tube and the tube was agitated manually two times during a 15-minute incubation at room temperature. Then 2 ml of the inactivated specimen was transferred to the test cartridge using a sterile disposal pipette. The cartridge was loaded into the Gene Xpert machine and an automatic process completed the remaining assay in 120 minutes. Interpretation of data from Xpert MTB/RIF test was software based.^17^ For samples whose results were invalid, a re-run of the test was carried out using a new cartridge. Error codes generated for error results were read off from the chart and the test repeated using a fresh, early morning sputum sample.

### Data analysis

All variables were described by proportion. The Chi-Square test was used to compare difference between independent groups as well as ratio analysis to determine the relative risk using confidence intervals P<0.05 was considered statistically significant.

### Ethical Approval

Ethical approval was obtained from Ethical Review Committees of each hospital. Patients voluntarily consented to participate in the study after adequate information about the nature of the study was provided.

## Results

Rifampicin resistance was detected in 88 out of 1300 Presumptive MDR-TB patients giving a prevalence of 6.8% (95% CI : 5.5–8.3). As shown in Table 1, more males (9.0%) were infected than females (4.2%) with rifampicin resistant tuberculosis (P = 0.036<0.05).

**Table 1:** Profile of RR-TB among pulmonary TB patients in Enugu between 1st April 2017 to 31st March 2019

Variable	No. Examined (%)	Rif Resistant (%)	Rif Susceptible (%)
Total	1300		
Male	702(54)	63(9.0)	639(91.0)
Female	598(46)	25(4.2)	593(95.8)

Age Group	Males	Female	Total	%
15–24	5	8	13	14.8
25–34	13	3	16	18.2
35–44	18	7	25	28.4
45–54	15	4	19	21.0
55–64	7	3	10	11.4
Above 65	5	0	5	5.7
Total	63	25	88	

Type of TB				
New	27	8	35	2.7
On treatment	36	17	53	4.1
Total	63	25	88	6.8

Rif Rifampicin; RR-TB---- Rifampicin resistant Tuberculosis

The clinical and socio-demographic characteristics of the RR-TB patients are presented in Table 2. Participants in the age group of 35–44 years had the highest rifampicin resistance (28.4%) (p<0.05). Among the patients, 35 (2.7%) were newly infected (treatment naive) whereas 53 (4.1%) were patients on anti-tuberculosis therapy (previously treated).

**Table 2:** Clinical and Socio-demographic characteristics of RR-TB among Pulmonary TB patients in Enugu Between 1st April 2017 to 31st March 2019

Variable	Total	RR-TB Positive	%	Relative risk 95% CI
Total	1300	88	6.8	6.8(5.5–8.3)
Male	702	63	9.0	1.36(1.18–1.56)
Female	598	25	4.2	
Age group (years)				
15–24	217	13	14.8	**1.9 (1.02–1.40)**
29–34	263	16	18.2	1.56
35–44	328	25	28.4	**1.20 (1.02–1.40)**
45–54	250	19	21.0	1.35
55–64	157	10	11.4	2.49
65 and above	85	5	5.7	2.49
Total	1300	88		
Type of TB Patient				
New	517	35	2.7	0.4
Previously treated	783	53	4.1	0.6
Total	1300			

RR-TB Rifampicin-resistant tuberculosis

The male group is the reference group with the highest risk of infection. Participants in the age group of 35–44 years is the reference group with the highest rifampicin resistance of 28.4% with very high risk relative to other age groups. Indication that MDR/RR-TB spread

## Discussion

MDR/RR-TB is a major public health problem which presents a new barrier to TB control worldwide. The spread of MDR/RR-TB is on the increase in the world in new and retreatment cases of patients suffering from TB^18^.

In this study, the prevalence of rifampicin resistant (RR-TB) was 6.8%. This result is similar to 5.9% obtained in Zambia2 but lower than 26.1% in Uttar Pradesh^1^. These high rates of rifampicin resistant MTB may be attributed to non-adherence to treatment regimens, differences in sampled population and/or poor TB control practices.

Males were predominantly infected with RR-TB. This trend is similar to other studies in Nigeria 12, India^19^ and Tanzania 20. Disparity in gender distribution of RR-TB could have been so due to the fact that men are more exposed to factors that lead to rifampicin resistance such as overcrowding in market places, poor adherence to treatment, smoking and alcoholism which make them more susceptible. This is also consistent with global trends in TB by gender^21^.

This study also revealed that in Enugu, the age group 35–44 years had the highest RR-TB and about 75% of the RR-TB patients were below 45 years old. Comparable results have been previously reported 1, 22, 23. In high TB burden countries, TB coincides with HIV prevalence in this age group. This may be the reason for a high prevalence of TB in this group.

Primary drug resistance was seen in 2.7% of the studied population. These are treatment naïve patients who have been infected with resistant Mycobacterium tuberculosis strains. This is an indication that MDR/RR-TB spread on the community could be on-going. This situation poses serious challenge to TB control. The National Tuberculosis and Leprosy Control Programme shift in TB algorithm to Xpert MTB/RIF as the initial diagnostic test instead of smear microcopy is therefore a step in the right direction.

Acquired resistance to RR-TB was observed in 4.1% of the patients. These were patients who develop resistance during therapy for tuberculosis (treatment failed patients). This prevalence of RR -TB among previously treated patients is consistent with 4.2% recorded in North-West Nigeria 22 but lower than 8.6% reported in Sagamu, Nigeria^24^ and 37.8% in India 19. In the presence of sub-lethal doses / inadequate treatment, mutant organisms resistant to anti-tuberculosis medicines emerge^25^. Other studies have established that previous treatment with anti-tuberculosis therapy is an important risk factor for tuberculosis resistance1 - worldwide^10^, ^26^, ^27^

## Conclusion

The prevalence of RR-TB was high in Enugu. Primary resistance in treatment naïve patients was also high. This study highlights that spread of MDR/RR-TB among young male patients in Enugu could be on-going.
